# Degradation and stabilization of gel electrolytes for quasi-solid Zn-air batteries

**DOI:** 10.3389/fchem.2026.1983516

**Published:** 2026-10-23

**Authors:** Manhui Wei, Zhuo Chen, Jianbin Li, Keliang Wang, Yue Peng, Zhouyang Xu

**Affiliations:** 1 Research Institute of Chemical Defense, Beijing, China; 2 School of Chemistry and Chemical Engineering, Huazhong University of Science and Technology, Wuhan, China; 3 School of Mechanical Engineering, Beijing Institute of Technology, Beijing, China

**Keywords:** degradation and stabilization, flexibility, gel electrolyte, ionic conductivity, quasi-solid Zn-air batteries

## Abstract

Gel electrolytes have emerged as the preferred electrolyte materials for quasi-solid Zn-air batteries, attributed to the high ionic conductivity and excellent mechanical flexibility. Nevertheless, the practical implementation faces multiple degradation challenges, including water loss, CO_2_ erosion, mechanical damage, thermal effect, and contact failure, which severely constrain cyclic lifetime and environmental adaptability. This review systematically examines the degradation mechanisms of gel electrolytes and the corresponding mitigation strategies, covering recent progress in water retention enhancement, anti-CO_2_ poisoning design, mechanical reinforcement, wide-temperature adaptability improvement, and Zn dendrite suppression. Current evidence indicates that single modification approaches are often insufficient to simultaneously enhance multiple performance. Future endeavors should therefore prioritize the design of gel electrolytes through multi-mechanism synergistic integration. Furthermore, a deeper understanding of structural evolution and failure behavior of gels under extreme conditions, combined with advanced characterization techniques, can guide the rational design and structural engineering of high-performance gel electrolytes, offering promise for advancing quasi-solid Zn-air batteries toward practical application.

## 1 Introduction

Energy crisis has spurred rapid advances in renewable energy technologies, and the batteries as core energy storage devices has achieved remarkable progress in recent years (Liu et al., 2018). The emergence of flexible electronic devices, such as foldable phones, smart bracelets and so on, has imposed new demands on energy storage systems, including mechanical flexibility, safety, and lightweight construction (Seneviratne et al., 2017). Many conventional lithium-ion batteries and some supercapacitors that rely on organic electrolytes pose safety risks such as flammability and corrosion, while the rigid structures also make it difficult to accommodate mechanical deformation (Jiao et al., 2023; Wei et al., 2026). Consequently, metal-air batteries have attracted considerable interest as promising candidates for next-generation flexible power sources, owing to the environmental friendliness, high safety, and inherent potential for flexibility (Xiao et al., 2023). Among different metal energy carriers, Zn-air batteries stand out due to the excellent stability, high theoretical energy density (1,086 W h/kg), abundant Zn resources, low cost, and facile recyclability, rendering the highly attractive in practical applications (Meng et al., 2024).

Electrolyte plays a pivotal role as the ionic transport medium in flexible energy storage devices. Traditional liquid electrolytes are susceptible to leakage under mechanical deformation, whereas all-solid-state electrolytes suffer from poor interfacial contact. Gel electrolytes combine the high ionic conductivity with the dimensional stability and excellent mechanical flexibility, and have therefore become a focal point in the development of flexible Zn-air batteries (Yang and Suo, 2018; Lu et al., 2020; Xia et al., 2023). Gels have been extensively studied in biomaterials since the first synthesis of crosslinked polyglycol monomethacrylate by Wichterle and LÍM (1960), and more recently have been introduced into Zn-air batteries (Misra and Siegel, 2002; Khutoryanskiy, 2007; Rong et al., 2018). Commonly used polymers include polyacrylamide (PAM), polyacrylic acid (PAA), and polyvinyl alcohol (PVA) (Santos et al., 2019; Chen et al., 2024). Besides, natural polysaccharide materials such as starch, cellulose, agar, carrageenan, and konjac gum have also been employed as the electrolyte. They are typically dissolved at elevated temperatures, and upon cooling, the dissolved linear molecular chains intertwine in a double-helix fashion to form a polymeric network (Othman et al., 2002; Cruz-Balaz et al., 2023). Owing to the abundant hydroxyl, aldehyde, and ether functional groups in polysaccharide molecules, hydrogen bonds can be formed, thereby enhancing water absorption and retention capacity, as well as the mechanical strength of the polymer network.

Nevertheless, gel electrolytes still face challenges in practical operation, including water loss, CO_2_ erosion, mechanical damage, thermal effects and contact failure, leading to the performance degradation and seriously impeding commercialization. This review aims to summarize the major degradation pathways of gel electrolytes, discuss typical strategies for enhancing the stability, and provide an outlook, with the goal of advancing the development of quasi-solid Zn-air batteries. Finally, the framework of this review is illustrated in Figure 1.

**FIGURE 1:**
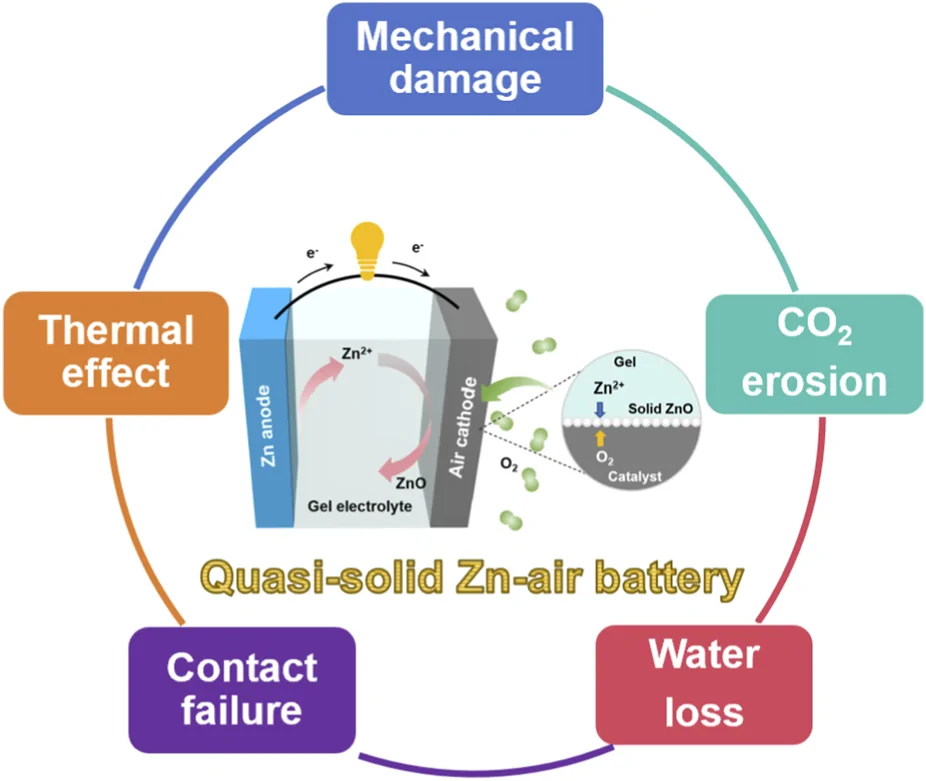
Framework of this mini review.

## 2 Degradation and stabilization of gel electrolyte

### 2.1 Water loss

Gel electrolytes are inherently aqueous systems, and the performance relies heavily on the water content within the electrolyte. Water molecules not only solvate ionic species but also participate in the hydrogen-bonding network that governs polymer chain mobility and ion transport. Once dehydration occurs, the ionic conductivity decreases and the interfacial charge transfer efficiency drops, leading to increased battery internal resistance and aggravated polarization. Moreover, a semi-open structure of Zn-air batteries exposes all components to flowing air, making water loss unavoidable. Unlike liquid electrolytes, quasi-solid Zn-air batteries using gel electrolytes cannot maintain sufficient water simply by adding a liquid reservoir. Therefore, enhancing the water retention capability of gel electrolytes has become a critical step toward the practical application of the batteries.

To mitigate the water loss of gel electrolyte, one effective strategy is to incorporate hygroscopic components into the polymer network to reinforce water-locking ability. The ionic liquid 1-butyl-3-methylimidazolium bromide has been introduced into the PAM gel, the water retention of the gel electrolyte is significantly improved owing to the hygroscopicity of the ionic liquid and the hydrogen-bonding network formed with water molecules and polymer chains (Zhang et al., 2025). As a result, the gel retains 81.2% of the initial water content after 50 h at 25 °C and 60% relative humidity, with an ionic conductivity of 340 mS/cm. Consequently, the assembled flexible Zn-air battery can achieve a lifetime of 433 cycles, a power density of 101.2 mW/cm^2^, and a specific capacity of 727.8 mA h/g.

Alternatively, constructing porous structures while incorporating highly water-retentive components can enhance the water adsorption and holding capacity synergistically. Sodium hyaluronate has been crosslinked with PAM, and SiO_2_ nanoparticles has been added as a pore-forming agent to fabricate a porous gel electrolyte (Li et al., 2025). The resulting composite gel exhibits excellent water adsorption capacity and effectively inhibits Zn dendrite formation, achieving a water retention rate of 80.6% after 96 h. Besides, the assembled flexible Zn-air battery can maintain 62% efficiency after 480 charge-discharge cycles, with a specific capacity of 735.5 mA h/g and a power density of 85.5 mW/cm^2^. Moreover, the stable operation for over 140 cycles at a high current density of 5 mA/cm^2^ also can be demonstrated.

### 2.2 CO_2_ erosion

The performance degradation in flexible Zn-air batteries can be triggered by the reaction of alkaline electrolyte with atmospheric CO_2_. In alkaline media, carbonate species exhibit low solubility in alkaline media and tend to precipitate at the cathode-electrolyte interface, thereby blocking oxygen diffusion channels and occupying active sites for the oxygen reduction reaction. Moreover, the mobilities of generated HCO_3_^−^ and CO_3_^2−^ are considerably lower than that of OH^−^, leading to a continuous decline in ionic conductivity of gel electrolyte and rapidly deteriorates battery performance. Consequently, anti-CO_2_ poisoning strategies are critical for ensuring stable operation of quasi-solid Zn-air batteries. Conventional liquid electrolyte systems can mitigate CO_2_ erosion by incorporating CO_2_ absorption filters or O_2_-selective membranes, as well as by periodically replacing the electrolyte (Huang et al., 2020). However, the structural constraints and application scenarios of flexible batteries have limited the applicability of the above approaches.

CO_2_ pre-fixation strategy can effectively address the issue. Inspired by the reversible reaction of CO_2_ with alcohols to form ionic compounds and the capture of CO_2_ in superbase media, CO_2_ is pre-fixed onto PVA chains by the strong organic base tetramethylguanidine (TMG), yielding a PVA-TMG gel polymer electrolyte (Zhou et al., 2021). The pre-fixed CO_2_ forms a protective layer on the electrolyte surface, which not only mitigates further attack by environmental CO_2_ but also significantly suppresses Zn dendrite growth and ZnO deposition, while enhancing ionic conductivity, water retention, and thermal stability. In a 22.7% CO_2_-rich atmosphere, the cycling stability of the battery using PVA-TMG gel electrolyte is improved by approximately 12-fold compared to pristine PVA.

By developing near-neutral gel electrolytes, the reaction between alkali and CO_2_ can be fundamentally avoid. In near-neutral media, the concentration of OH^−^ is low, so CO_2_ dissolution does not lead to significant carbonate precipitation, thereby suppressing CO_2_-induced degradation at the source. A PVA gel has been immersed in chloride-based solution with low ZnCl_2_ concentration and fixed 2.8 M NH_4_Cl, which maintains the high water content and delivers a power density of 15.3 mW/cm^2^ (Lai et al., 2022). Meanwhile, a high ZnCl_2_ concentration is found to suppress Zn anode passivation caused by precipitation of Zn(NH_3_)_2_Cl_2_ and Zn_5_(OH)_8_Cl_2_·H_2_O. Under optimized conditions (0.6 M ZnCl_2_/2.8 M NH_4_Cl), the assembled gel-based Zn-air battery can achieve a cyclic lifetime exceeding 100 h.

### 2.3 Mechanical damage

The primary advantage of flexible Zn-air batteries lies in the ability to withstand complex deformations such as bending, folding and twisting. If the gel electrolyte cracks or fractures during such processes, the electrode-electrolyte interface and internal ion transport pathways will be compromised, leading to the battery failure. Therefore, high mechanical strength and shape recovery capability are essential properties for the gel electrolytes. Mechanical performance of the gels is governed by intermolecular interactions within the polymer network, including hydrogen bonds, covalent bonds, and ionic bonds. A greater variety and higher density of such bonds generally result in a more robust network structure, as they can effectively dissipate energy and resist crack propagation.

Introducing a second polymer network to enrich the types and number of functional groups and increase hydrogen bond density is a widely adopted strategy for enhancing gels mechanical properties of gels. A dual-network alkaline gel electrolyte is proposed by combining *in situ* polymerized covalently crosslinked PAM with freeze-thaw polymerized physically crosslinked PVA (Zhang et al., 2023). Benefiting from the synergistic effect of the dual-crosslinked network and hydrogen bonding, the gel can achieve an ionic conductivity of 309.9 mS/cm, a mechanical toughness of 0.69 MJ/cm^3^, and a maximum fracture stress of 498.4 kPa with a tensile strain of 237.85%, substantially outperforming single-network PVA (≈86.1 kPa) and PAM (≈107 kPa).

Embedding a high-strength three-dimensional skeleton into the gel represents another effective approach. Agar is incorporated into a porous hydrophilic melamine foam scaffold, which has been upgraded to an enhanced-performance gel electrolyte (Zuo et al., 2023). Owing to the flexibility of the melamine skeleton, the mechanical performance of the gel is improved by 365.3% compared to pristine agar. The continuous skeleton can bear and distribute external stress, thereby preventing stress concentration and crack propagation within the gel matrix. Moreover, the gel sustains no structural damage after 100 cycles of 180° bending and withstands a compressive force of 138.2 N without fracturing. Meanwhile, the porous structure effectively alleviates the passivation of Zn anode, enabling the assembled battery to deliver a power density of 126 mW/cm^2^ and an areal capacity of 24 mA h/cm^2^.

### 2.4 Thermal effects

Temperature adaptability is a critical challenge for the application of flexible Zn-air batteries in extreme environments. At low temperatures, gel electrolytes suffer from reduced ionic conductivity and surface icing, because water molecules freeze and disrupt continuous ion-transport pathways. At high temperatures, rapid water evaporation causes dehydration of the gel, leading to deteriorated mass and charge transfer and aggravated polarization (Yang et al., 2021; Hou et al., 2023). Therefore, enhancing the wide-temperature stability of gel electrolyte is an essential prerequisite for ensuring efficient battery operation.

To mitigate low-temperature performance degradation, incorporating functional additives into the gel electrolyte to reduce water activity and inhibit ice crystal formation has proven effective. 2 M Zn(ClO_4_)_2_ has been incorporated into a dual-network gel composed of carboxymethyl chitosan and PAM (Huang et al., 2022). Through the Hofmeister effect, ClO_4_^−^ can form weak hydrogen bonds with water molecules and the polymer matrix, disrupting the strong hydrogen-bond network among water molecules and thereby significantly lowering the freezing point of the electrolyte. As a result, the obtained gel maintains the excellent electrochemical performance at −30 °C, and the assembled symmetric Zn batteries achieves stable cycling for 1,200 h at this temperature, demonstrating outstanding low-temperature tolerance.

Microphase separation offers a promising approach to overcoming the challenge of wide-temperature adaptability of gel electrolyte. Cellulose nanofibers has been utilized to regulate the microphase-separated structure of the gel electrolyte, achieving structural-functional partitioning between polymer-rich and solvent-rich domains, which enables an ionic conductivity of 30.95 mS/cm in the non-alkaline system and rises to 318.21 mS/cm in the alkaline system (Wang et al., 2025). More importantly, the gel electrolyte can maintain stable charge-discharge performance across a wide temperature range from −40 °C to 80 °C, effectively overcoming the dual constraints of low-temperature freezing and high-temperature water loss on battery performance. The microphase-separated structure not only provides a viable pathway to mitigate thermally induced performance degradation but also offers a new direction for the designing electrolytes for wide-temperature flexible Zn-air batteries.

### 2.5 Contact failure

Zn dendrite growth is a critical factor that reduces electrode surface area, causes loss of active material, and aggravates polarization, thereby severely limiting the cyclic lifetime of battery. Although gel electrolytes can suppress dendrite formation through mechanical confinement, the interfacial contact with the Zn anode is less stable than that in liquid electrolytes. This poor contact tends to induce uneven distributions of electric field and ion concentration, creating localized current hotspots that promote preferential Zn deposition at protrusions and exacerbate dendrite propagation. The resulting increase in interfacial resistance paradoxically accelerates Zn dendrite growth (Liu et al., 2016; Yang et al., 2022). Therefore, enhancing the interfacial affinity of gel electrolytes is an important direction for improving mass and charge transfer performance of the battery.

Constructing a self-adaptive interface can enable the gel to dynamically conform to changes in the Zn anode, thereby homogenizing current and ion distribution. Sodium alginate has been introduced into a P (AM-co-AA) network to obtain an interpenetrating gel electrolyte (Yuan et al., 2025). Leveraging the high hydrophilicity and zincophilicity, the gel electrolyte preferentially adsorbs onto the Zn anode surface, effectively suppressing dendrite growth and the formation of zincate by-products. Finally, symmetric Zn batteries using the gel electrolyte can achieve the stable cycling for 800 h at 2 mA/cm^2^.

Regulating the solvation structure of Zn^2+^ through functional group coordination can weaken the binding between water molecules and Zn^2+^, thereby promoting uniform Zn deposition and inhibiting side reactions. A ternary deep eutectic solvent system of acetamide/ZnCl_2_/H_2_O has been introduced into PAA polymer to prepare a composite gel electrolyte (Liu et al., 2024). The polar groups C=O and -NH_2_ in acetamide coordinate with Zn^2+^, reducing the interaction between Zn^2+^ and water molecules as well as the ionic bonding in ZnCl_2_, which endows the gel a wide electrochemical window and low corrosivity, enabling highly reversible dendrite-free cycling. As a result, the Zn-air battery assembled with the gel electrolyte achieves a cyclic lifetime of 900 h.

## 3 Conclusions and outlook

Gel electrolytes, owing to the high ionic conductivity and excellent flexibility, have become the preferred electrolyte materials for flexible Zn-air batteries, yet still face serious challenges including water loss, CO_2_ erosion, mechanical damage, insufficient temperature adaptability, and contact failure. For addressing the issues, a series of strategies have been developed, such as dual-network construction, functional additive incorporation, three-dimensional skeleton compositing, CO_2_ pre-fixation, interfacial affinity regulation and so on, which have achieved notable progress in enhancing water retention, mechanical flexibility, and wide-temperature stability. Nevertheless, a single modification strategy is often insufficient to simultaneously improve multiple performance metrics. The overall performance of gel electrolytes still needs further enhancement.

In the future, the following aspects can be considered, as summarized in Figure 2. Firstly, synergistic modification strategies that integrate water retention, anti-CO_2_, mechanical reinforcement, and interface optimization into a single system should be explored to overcome the trade-offs among different performance metrics. Secondly, the structural evolution and failure mechanisms of gels under extreme conditions should be further elucidated to provide theoretical guidance for wide-temperature operation. Thirdly, *in-situ* and *ex-situ* characterization techniques should be developed to monitor the microstructural changes of gels during operation in real time, thereby establishing a quantitative correlation between structure and performance. Moreover, the structure-performance co-optimization of green and sustainable materials should be considered to facilitate the scale-up and commercialization of Zn-air batteries. In summary, through rational material design and structural regulation, gel electrolytes are expected to advance quasi-solid Zn-air batteries toward practical application.

**FIGURE 2:**
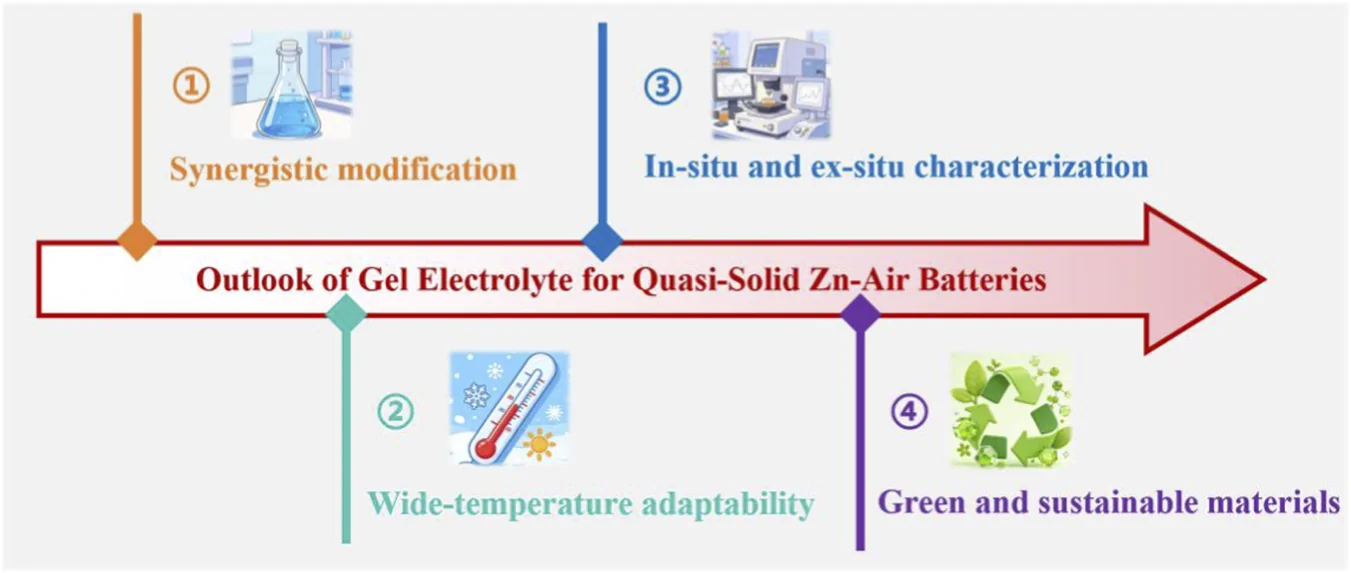
Outlook of this mini review.
